# Method for Rationalising the Operational Process of a Manual Motorway Toll Collection System

**DOI:** 10.3390/s21103497

**Published:** 2021-05-17

**Authors:** Zbigniew Kasprzyk, Mirosław Siergiejczyk, Mariusz Rychlicki

**Affiliations:** Faculty of Transport, Warsaw University of Technology, Koszykowa 75, 00-662 Warsaw, Poland; miroslaw.siergiejczyk@pw.edu.pl (M.S.); mariusz.rychlicki@pw.edu.pl (M.R.)

**Keywords:** toll collection process, application supporting the toll collection process, operation of the manual toll collection system

## Abstract

The article presents the verification of a developed method of rationalising the process of the operation of a manual toll collection (MTC) system on the basis of current operation tests. The review of the state of art and research on the operation of the manual toll collection system prompted the authors to develop their own method of rationalising the operation process of the manual toll collection system. The method enabled the development of an original application facilitating the process of manual toll collection system. The application makes it possible to determine the key indicator—the readiness index for the employed operation strategy of the analysed toll collection station and the readiness index for the employed operation strategy of the analysed group of toll collection stations. Additionally, the application enables an analysis of the capacity and the service assessment by motorway users. The use of the software system together with the developed method was implemented on a real toll plaza. This action allowed for the analysis and implementation of an adequate MTC service strategy. The results of the research and analysis are presented in the summary and conclusions.

## 1. Introduction

The toll collection process should be smooth and ensure the greatest possible continuity of the transport service and an adequate quality of a toll road user service. Toll road users accept the fact of collecting fees for passage, provided that they are guaranteed relevant comfort of travelling on national roads, and thus, ensuring the handling of paying users, together with the continuity of the transport service. This heterogeneity of the toll collection system forces an administrator of a given motorway segment to use two systems of manual toll collection along with an electronic one, which additionally increases the system operation costs and makes it impossible to ensure the same quality of payment services for each vehicle type.

In the light of the above, it can be concluded that the reliability of a highway toll collection system determines the continuity of the transport service and the revenue from tolls on national roads. Achieving a high level of reliability of a highway toll collection system is particularly important in places where the fast and safe transport of people and goods is of large importance. An interrupted operation of a highway toll collection system results in vehicle traffic congestion and a termination of transport service continuity. 

Motorway toll collection systems operate in various operating conditions, and their correct functioning is determined not only on the reliability of individual system elements, but also on the applied operational strategies. Given the above, there is a need to analyse the reliability and the operation process of a manual toll collection (MTC) system and to propose an efficient method of its improvement.

The introduction of the National Toll Collection System in Poland created the need to integrate manual and electronic toll collection systems. The National Toll Collection System enables to collect tolls both electronically and manually, depending on the type of vehicle. In accordance with the provisions of the Terms of Reference for a limited tender of the National Toll Collection System, including activities related to electronic toll collection (ETC), all vehicles with a permissible total weight below 3.5 tons are required to pay the toll on state motorways using the MTC system. The obligation to pay the electronic fee applies to vehicles with a maximum permissible weight of more than 3.5 tons and buses. Therefore, the use of MTC systems must be carried out simultaneously with ETC on the toll road network in Poland. MTC is a process that, from the point of view of the road user, disrupts the transport process and measurably contributes to the extension of its execution in time. Therefore, it is advisable to conduct appropriate tests, the purpose of which is, inter alia, to determine the reliability of the MTC system. This analysis of the reliability of the MTC system is aimed at obtaining information on the performance of technical objects during operation. The obtained indices will allow the authors, using the proprietary application, to evaluate the actual analysed system, and thus to define the strategy of operation so that it meets the requirements specified in norms and normative regulations. To assess the reliability of the MTC system, the system availability index was used, which is a numerical measure of its reliability evaluation.

The literature contains numerous papers dealing with the theory of reliability and the operation of technical systems. Examples of publications in the field of studying the reliability and operation of technical facilities include [1,2,3,4,5,6,7,8,9,10,11,12,13]. The article [2] presents considerations regarding the readiness of the motorway emergency communication system, which is an indispensable element of the motorway equipment. The author presents an approach from the point of view of network readiness to provide emergency communication services. In [3], a method based on graph theory and Boolean function for assessing reliability of mechanical systems is proposed. The procedure for this approach consists of two parts. By using the graph theory, the formula for the reliability of a mechanical system that considers the interrelations of subsystems or components is generated. The combination of graph theory and Boolean function provides a way to evaluate the reliability of a mechanical system. However, this study does not consider the reliability and readiness of the entire toll collection system on the motorway. These works discuss the general analysis of the reliability and operation of technical objects or present an analysis of specific cases of electromechanical devices. However, there is a lack of an analysis in terms of toll collection systems, which are complex technical systems operating in specific conditions. An attempt at such an analysis of the reliability and readiness of the entire toll collection system on the motorway was presented in [14].

The reliability evaluation of an MTC system generates the problem of constructing an appropriate model representing the operation of an actual system. The transport domain contains numerous elaborations regarding the modelling of transport systems and processes. The examples in this field are the following papers: [4,15,16,17]. The manner of formally describing transport systems of the above group of research papers depends on the system expansion degree, the modelling objective, as well as the type of objects and processes being modelled. The modelling process utilises analytical methods that contain mathematical models taking into account the theory of information [2,8,18], the theory of reliability [1,5,8,10,11,13,15,17,19,20,21], the theory of mass service [22,23], the theory of stochastic processes [1,5] and the theory of graphs and networks [5,15,18,21]. These methods are used at each of the transport system modelling stages.

The analysis of transport system functioning made the authors of many papers to address the issue of reliability of transport systems and devices, which are the components thereof [2,21]. The example section of the paper [24] on the reliability of technical objects contains a discussion regarding transport systems. The reliability analysis of transport systems quite often shows that it is necessary to use a method of estimating their reliability parameters. In many cases, such a situation occurs when there are no elaborations on the behaviour of the analysed system or lack of knowledge in terms of the correct time distribution of operation for objects, which are the components of a transport system. An example of such systems are toll collection systems. Studies regarding the issue of reliability of transport systems, and toll collection systems in particular, using methods of estimating reliability and serviceability of technical objects are shown, among others, in [14,25,26,27].

There are many works [28,29] related to the analysis of fuel consumption by vehicles participating in the toll collection process. For example, the paper [29] deals with a very important topic concerning the analysis of the fuel consumption of vehicles involved in the tolling process in electronic and manual tolling systems in China, where traffic is very heavy. The paper presents a vehicle emission model based on trees, considering how traffic at toll plazas on motorways can cause severe pollution due to increased emissions from vehicles stopping and starting. Road emission data and vehicle usage data were obtained from two different toll plazas on motorways. The results of this study can facilitate the estimation of vehicle emissions and improve the operation of electronic toll collection (ETC) on motorways. An appropriate strategy for the operation of toll collection stations is also related to the reliability aspect of these systems and the analysis of the capacity of the toll collection systems. This work does not address the topic of reliability analysis together with the analysis of the capacity of the toll collection systems, which seem to be very interconnected.

The analysis of the literature also made it possible to raise important aspects related to the COVID-19 pandemic and its impact on the development of transport systems. For example, [30] analysed the short-term effects on the transport system of various strategies adopted by the Colombian government and local authorities to contain the spread of COVID-19.

The above papers concern the assessment of technical systems not related to the toll collection process. They mainly regard fuel consumption by vehicles participating in toll collection in relation to COVID-19 restrictions. Each of the presented assessment methods takes into account the specific operating conditions of technical systems, which are often unusable when assessing other systems. Due to the complexity of modern technical systems, it is often necessary to develop a unique reliability assessment method for each of the analysed systems. There are no specific practical solutions in the literature regarding the development of a toll collection strategy in MTC systems. 

The authors of this article focused on the verification of a developed method, based on actual operational studies, which are the current basis for improving technical systems and their maintenance process.

## 2. A Developed Method for Rationalising the Operational Process of a Manual Motorway Toll Collection System

The purpose of the reliability analysis carried out in [14] was to obtain the forecasted reliability parameters, which were used to control the reliability of the MTC system in the form of a grouping of toll collection stations. The reliability assessment of the system showed that it does not meet the reliability and availability requirements of the MTC system. Therefore, there is a need to analyse the operation process of the MTC system, which defines all technical and organisational activities relating to the system at all stages of its existence, from production to decommissioning. Technical activities, through the proper use of the system, are aimed at ensuring its greatest possible readiness. Similarly, organisational activities through appropriate service and supply are aimed at maintaining the required readiness of the system. Appropriate operation of the system will enable the performance of activities connected to preparing or maintaining the system in a state of readiness with the required reliability. The analysis of the operation process of the MTC system presented below helps define the system operation strategy in the form of appropriate organisational activities aimed at maintaining the system in the required reliability and readiness state.

### 2.1. Assumptions for Modelling the Operation Process of the Manual Toll Collection System

Both the performed characteristics and functional analysis of the MTC system at work [14] indicate that the reliability of its elements is the factor shaping the operation of the MTC system. The elements of the system are toll collection stands located in a grouping of stands on the toll plaza. Each of the toll collection station operates independently of the others in the system, the stations are connected in a parallel manner. Such an operation of the MTC system enables the analysis of the operation process of a single toll collection station as an independently operating element of the entire system. Therefore, the model of the operation process of the MTC system was developed on the example of a toll collection station that independently implements the toll collection process in a grouping of positions in the system. Only the current state of the system has a direct impact on changing the state of the MTC system in the future. A convenient class of analytical models that can be applied in the described case is the Markov process for which [25]:

(1) $$P\left(\xi \left(t_{n}\right)<x_{n}/\xi \left(t_{n-1}\right)=x_{n-1},\ldots ,\xi \left(t_{0}\right)=x_{0}\right)=P\left(\xi \left(t_{n}\right)<x_{n}/\xi \left(t_{n-1}\right)=x_{n-1}\right)$$

for any string of parameters $t_{0}<t_{1}<\cdots <t_{n-1}<t_{n}$.

Equation (1) shows that the state of the process at a moment is not directly influenced by the states it was in at times. Only the state in which the process is in moments has a direct impact. For Markov processes, only the present determines what will happen in the future, and the past (history) has no influence.

The operation of the MTC system allows us to state that examining this process only in selected moments of time may be insufficient. In the case of processes with a continuous time parameter, the analysis of the system operation process will be more accurate, which will enable a rational definition of the operation strategy of the analysed system.

In connection with the above, it was concluded that the Markov process model discrete in states and continuous in time, will be the appropriate type of model to describe the operation process of the manual toll collection system. It is characterised by a finite or countable number of process states where the parameter t (time) changes continuously and the transition from one state to another is possible at any time.

The model of the operation process of the toll collection station that independently implements the toll collection process in the grouping of toll collection stations was made with the following assumptions:

– times of the process being in the distinguished operating states are random variables with exponential distributions,
– the probability of two or more damage to the toll station modules at the same time is close to zero,
– during operation, the state of the system changes randomly over time.

The following group of indicators was defined to describe the operation process of the toll collection station:

– stationary probabilities of operating process states,
– ready indicator.

The distinguished indicators do not exhaust all that can be used to describe the operation process of the manual toll collection system. However, they seem sufficient for a practical assessment of the operation process of the tested system.

### 2.2. Model and Description of the Operation Process of the MTC System

During operation, technical systems are in various operational states. In the exploitation theory, there are three subsets of the operational states of technical systems [4,5,6]:

– a subset of pending states,
– a subset of usage states,
– a subset of servicing states.

An example of a subset of the standby states of technical systems are [4]: storage, standstill between successive states of use, etc. In transport systems, different states of use can be mentioned: laden mileage, loose mileage, or the system effective operating state. The subset of maintenance states includes states in which various types of service are performed, such as: supply, serviceability control, preventive repairs, current (renovation) repairs.

Taking into account the set of functional states, as well as the serial functional structure of the toll collection station located in the grouping of stations belonging to the manual toll collection system, the following operating states of the station were distinguished:

– *S*_1_-the state of effective work,
– *S*_2_-state of waiting for repair,
– *S*_3_-repair status.

The states of *S_i_* form a state space defined by the set *S*:

(2) $$S=\left\{S_{1}{,S}_{2}{,S}_{3}\right\}$$

**Figure 1:**
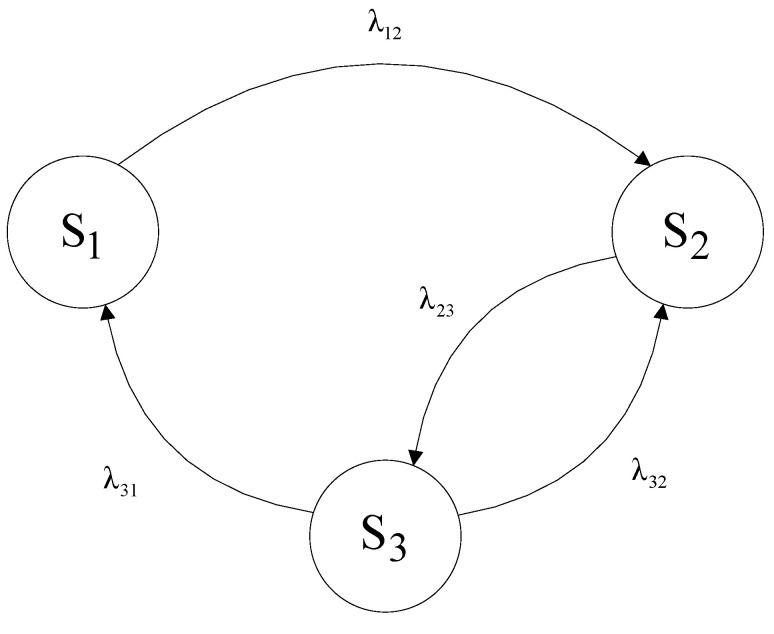
Graph of the toll station operation process, taking into account the intensity of transitions. Source: own study.

The graph of the operation process of the toll collection station, taking into account the intensity of state transitions, is shown in Figure 1. State transitions are characterised by the process intensity functions $\lambda_{ij}$. The residence times of the process in the state space *S* have an exponential distribution.

During the operation of the toll collection station, two basic phases were distinguished in terms of the scope of its operation. This is the use phase, which includes the *S*_1_ state of the toll station operation process and the service phase, which includes the *S*_2_ and *S*_3_ states shown in Figure 1.

The use phase is the process in which the station is used for its intended purpose, that is, it performs the efficient work of collecting the toll and carries out tasks within its limits. The operating phase of the station consists in carrying out activities related to the preparation or maintenance of the station ready for use. These activities involve the maintenance of the stand in order to prepare for repair in the event of damage to any module of the stand, and the repair of the damaged module itself. The toll collection stand, which is an integral part of the MTC system, while in the *S*_1_ state of effective work, can go into the state of awaiting repair *S*_2_ with the intensity of transition $\lambda_{12}$. From the state of pending repair, the toll plaza may move with intensity $\lambda_{23}$ to the state of repair *S*_3_. Being in the *S*_3_ state, it is possible to go to the *S*_1_ state (with the transition intensity $\lambda_{31}$) and the *S*_2_ state (with the transition intensity $\lambda_{32}$). The transition from the state *S*_3_ to the state *S*_1_ takes place when the toll collection stand is restored. The transition from state *S*_3_ to state *S*_2_ takes place while waiting for spare parts. The system of the Kolmogorov–Chapman equations for the toll station operation process shown in Figure 1 is as follows:

(3) $$\{\begin{array}{l} -\lambda_{12}\cdot P_{1}+\lambda_{31}\cdot P_{3}=0\\ -\lambda_{23}\cdot P_{2}+\lambda_{12}\cdot P_{1}+\lambda_{32}\cdot P_{3}=0\\ -\lambda_{31}\cdot P_{3}+\lambda_{23}\cdot P_{2}-\lambda_{32}\cdot P_{3}=0 \end{array}$$

One of the equations was replaced with the normalization condition, obtaining:

(4) $$\{\begin{array}{l} -\lambda_{12}\cdot P_{1}+\lambda_{31}\cdot P_{3}=0\\ \lambda_{12}\cdot P_{1}-\lambda_{23}\cdot P_{2}+\lambda_{32}\cdot P_{3}=0\\ P_{1}+P_{2}+P_{3}=1 \end{array}$$

Using the matrix notation, the system of equations has the form:

(5) $$\underset{A}{\underbrace{\left[\begin{array}{ccc} -\lambda_{12} & 0 & \lambda_{31}\\ \lambda_{12} & -\lambda_{23} & \lambda_{32}\\ 1 & 1 & 1 \end{array}\right]}}\cdot \underset{P}{\underbrace{\left[\begin{array}{c} P_{1}\\ P_{2}\\ P_{3} \end{array}\right]}}=\underset{B}{\underbrace{\left[\begin{array}{c} 0\\ 0\\ 1 \end{array}\right]}}$$

where:

***A***-matrix of coefficients,
***P***-column matrix (stationary system probabilities),
***B***-column matrix of intercepts.

If we assume that the stationary probabilities of the operating process states of the toll collection station are determined by the following formulas:

(6) $$P_{1}=\frac{\lambda_{31}\cdot \lambda_{23}}{\lambda_{12}\cdot \lambda_{31}+\lambda_{12}\cdot \lambda_{23}+\lambda_{12}\cdot \lambda_{32}+\lambda_{31}\cdot \lambda_{23}}\;P_{2}=\frac{\lambda_{12}\cdot \lambda_{31}+\lambda_{12}\cdot \lambda_{32}}{\lambda_{12}\cdot \lambda_{31}+\lambda_{12}\cdot \lambda_{23}+\lambda_{12}\cdot \lambda_{32}+\lambda_{31}\cdot \lambda_{23}}\;P_{3}=\frac{\lambda_{12}\cdot \lambda_{23}}{\lambda_{12}\cdot \lambda_{31}+\lambda_{12}\cdot \lambda_{23}+\lambda_{12}\cdot \lambda_{32}+\lambda_{31}\cdot \lambda_{23}}$$

So:

(7) $$P_{1}=\frac{\lambda_{31}\cdot \lambda_{23}}{\lambda_{12}\cdot \left(\lambda_{31}+\lambda_{23}+\lambda_{32}\right)+\lambda_{31}\cdot \lambda_{23}}\;P_{2}=\frac{\lambda_{12}\cdot \left(\lambda_{31}+\lambda_{32}\right)}{\lambda_{12}\cdot \left(\lambda_{31}+\lambda_{23}+\lambda_{32}\right)+\lambda_{31}\cdot \lambda_{23}}\;P_{3}=\frac{\lambda_{12}\cdot \lambda_{23}}{\lambda_{12}\cdot \left(\lambda_{31}+\lambda_{23}+\lambda_{32}\right)+\lambda_{31}\cdot \lambda_{23}}$$

The values of the stationary probabilities (7) are determined by the intensity of the transitions.

The availability ratio of the toll collection station described by the operating conditions graph presented in Figure 1 will be equal to the probability of the station being in the effective operating state *S*_1_:

(8) $$A_{\mathrm{ST}\_E}=P_{1}=\frac{\lambda_{31}\cdot \lambda_{23}}{\lambda_{12}\cdot \left(\lambda_{31}+\lambda_{23}+\lambda_{32}\right)+\lambda_{31}\cdot \lambda_{23}}$$

To determine the value of the toll collection station availability ratio, defined by the Equation (8), the following values were adopted, specified in the requirements for the reliability and availability of toll collection systems specified in the literature [19,27]:

– the average time between subsequent damage to a single toll collection station is MTBF=4380 h which corresponds to a damage intensity of $\lambda_{12}=2283\times 10^{-4}{\;h}^{-1}$,
– the total average repair time for a single toll collection station is MTTR=2 h, where $\lambda_{23}=1{\;h}^{-1}$ are the intensities of waiting for a repair and $\lambda_{31}=1{\;h}^{-1}$ are the repair intensities,
– the waiting time for replaceable modules is 30 min, that is, the intensity of waiting for spare parts $\lambda_{32}=2{\;h}^{-1}$, factor Ψ defining the demand for replaceable modules of a toll collection station.

For such adopted assumptions, the value of the readiness index determined by the relation was obtained:

(9) $$A_{\mathrm{ST}\_E}=\frac{\lambda_{31}\cdot \lambda_{23}}{\lambda_{12}\cdot \left(\lambda_{31}+\lambda_{23}+\lambda_{32}\right)+\lambda_{31}\cdot \lambda_{23}}=0.9990876$$

Equation (9) also allows to determine the impact of the change in the value of the repair intensity of the toll collection station on the value of the availability ratio. The toll station is in the subset of the servicing states with the probability determined by the following relationship:

(10) $$P_{2}+P_{3}=\frac{\lambda_{12}\cdot \left(\lambda_{31}+\lambda_{32}\right)}{\lambda_{12}\cdot \left(\lambda_{31}+\lambda_{23}+\lambda_{32}\right)+\lambda_{31}\cdot \lambda_{23}}+\frac{\lambda_{12}\cdot \lambda_{23}}{\lambda_{12}\cdot \left(\lambda_{31}+\lambda_{23}+\lambda_{32}\right)+\lambda_{31}\cdot \lambda_{23}}=0.0009124$$

Equation (10) also allows to determine the impact of the change in the value of the repair intensity of the toll collection station on the value of the probability of being in a subset of servicing states. The analysis of the presented model of the operation process of the toll collection station allowed to determine the dependencies enabling the determination of the value of being in the following states: effective work, waiting for repair and repair.

## 3. Verification of a Developed Method for Rationalising the Operational Process of a Manual Highway Toll Collection System Based on Actual Operational Studies

In practice, the correct organisation of the stock of exchangeable elements is of great importance, because the amount of stocks has a significant impact on the process of technical object renovation. The lack of appropriate elements may completely prevent the renovation of the object or significantly extend its repair time, when additional time is needed to look for appropriate replaceable elements. Such a situation is particularly undesirable in the case of toll collection systems, where any downtime in the proper operation of the toll collection station may lead to dangerous situations in the form of traffic congestion. It is extremely important to properly determine the stock of exchangeable modules and to develop an appropriate system maintenance strategy.

The method of rationalising the operation process of a MTC system can be verified based on the example of actual operational studies, which are currently the basis for improving the design of technical systems and their maintenance process. They enable obtaining reliable information necessary for controlling the operation process, proper service facilities organisation, and to predict and determine the operation costs of a toll collection system.

Operational studies of a MTC system in a station were conducted over a period of 6 months, which corresponds to 4380 h. The test period was determined based on the requirements in the field of reliability and availability of MTC systems presented in the literature [19,27]. 

The studies involved estimating the intensity of damage to individual replaceable modules, with the values shown in Table 1. 

**Figure 2:**
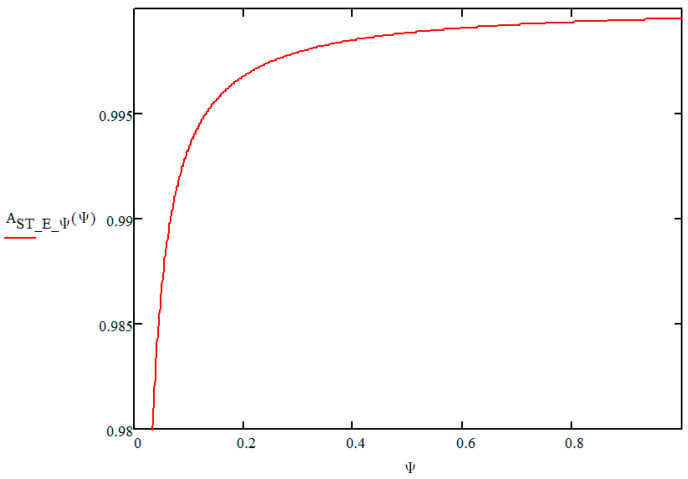
Graph depicting the impact of the number of replaceable modules of a manual toll collection station on its readiness index value. Source: own study.

Figure 2 shows a graph depicting the impact of the number of replaceable modules of a toll collection station on its readiness index value based on the aforementioned adopted operational data. Factor Ψ defining the demand for replaceable modules for a station adopts a value from the range of Ψ∈0,1. In order to guarantee the required readiness index value of a toll collection station specified in the reliability and availability requirements of MTC systems, the value of factor Ψ=1, that is according to the method described in paper [14] the following was obtained:

(11) $$A_{{\mathrm{ST}}_{E_{\Psi }}}\left(\Psi =1\right)=\frac{\Psi \lambda_{31}\lambda_{23}}{\lambda_{12}\lambda_{23}\lambda_{12}\lambda_{32}+\Psi \lambda_{12}\lambda_{31}-\Psi \lambda_{12}\lambda_{32}+\Psi \lambda_{31}\lambda_{23}}=0.9995436$$

According to the methodology in [26] regarding the rationalisation of the operation process of a MTC system based on the station, it was concluded that the total number of replaceable modules of the i-type needed to ensure the reliability of a toll collection station was 26 replaceable modules. Table 2 shows the number of replaceable modules at a toll collection station for the determination of an appropriate readiness index *A*_ST_ for the studied station, under predicted damage intensities of individual modules shown in Table 1.

A factor Φ is introduced that determines the actual expenditure on the replaceable modules needed for the correct operation of a toll collection station. It determines a linear relationship between the value of the actual expenditures allocated to the replaceable modules and the cumulative expenditures on the replaceable elements needed for the correct operation of a toll collection station.

The operational studies of a MTC system took into account the estimated intensities of individual replaceable module damage, the values of which are shown in Table 1 and estimated cumulative expenditure on replaceable elements needed for the correct operation of a toll collection station. The following initial conditions resulting from the values were adopted, specified in the requirements for the reliability and availability of toll collection systems specified in the literature [19,27].

**Figure 3:**
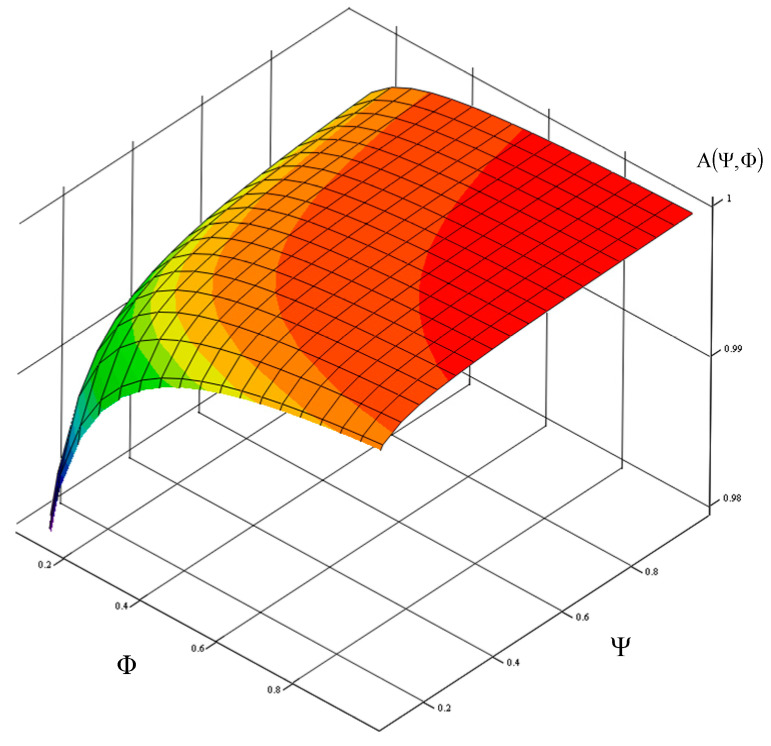
Graph showing the impact of a toll collection station replaceable module demand index and the index on expenditure on replaceable modules, on the value of its readiness index. Source: own study.

**Figure 4:**
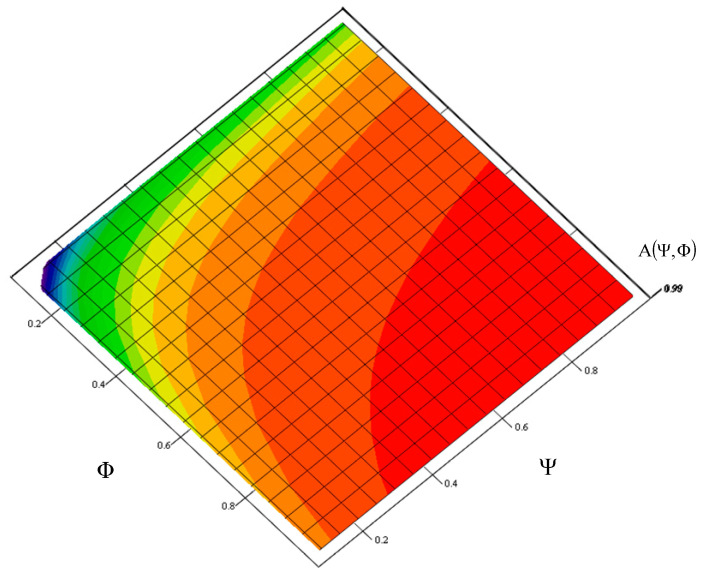
Graph showing the impact of a toll collection station replaceable module demand index and the index on expenditure on replaceable modules, on the value of its readiness index. Source: own study.

For these adopted operating data, Figure 2, Figure 3 and Figure 4 show a graph of the impact of the Ψ index of a toll collection station replaceable module number and the Φ index of the actual expenditure on replaceable modules needed for the correct operation of a toll collection station on the value of the toll collection station readiness index. It is a graphically illustrated criterion function (1.1). The Ψ index, which defines the demand for replaceable modules for a station adopt a value from the Ψ∈0,1 range, and the Φ index, which defines the actual expenditure adopts a value from the Φ∈0,1 range.

In order to guarantee the required readiness index value of a toll collection station specified in the reliability and availability requirements of MTC systems, the value of factor 

Ψ = Φ = 1, that is according to the relationship (11), the following was obtained:

(12) $$A\left(\Psi =\Phi =1\right)=\frac{\Phi \Psi \lambda_{31}\lambda_{23}}{\lambda_{12}\lambda_{23}+\Phi \lambda_{12}\lambda_{32}+\Phi \Psi \lambda_{12}\lambda_{31}-\Phi \Psi \lambda_{12}\lambda_{32}+\Phi \Psi \lambda_{31}\lambda_{23}}=0.9995436$$

Table 3 shows the number of replaceable modules necessary to ensure an appropriate value of the readiness index AST under factor Φ, which defines the actual expenditure on replaceable modules needed for the correct operation of a toll collection station and factor Ψ, which defines the demand for replaceable modules of a toll collection station.

For example, the case of the data above and cumulative expenditure C on the replaceable elements needed for the correct operation of a toll collection station at a level of PLN 500,000 resulted in a list of the number of toll collection station replaceable modules needed to ensure an adequate value of a station readiness index. The summary is shown in Table 4.

In the event of a demand for 26 replaceable modules needed to ensure the required readiness index of a toll collection station, and the actual expenditure on replaceable modules being lower than the expenditure required for the correct operation of a toll collection station, there is a possibility to determine the maximum value of the readiness factor A_ST_ for a toll collection station and vice versa. The developed operational strategy of maximising the readiness factor, which takes into account the demand for replaceable modules and the actual expenditure on the modules in the course of the system operation enables accurate organisation of toll collection station replaceable element stock.

The developed operation process rationalisation method presents issues associated with distinguished operating states of a studied, MTC station. The demand for spare parts of a toll collection station was conducted and a factor taking into account expenditure on the parts was introduced. This enabled the development of an operational maximization strategy for the readiness factor, which takes into account the demand for replaceable modules and the actual expenditure allocated to the modules within the operation process of a system. This made it possible for the decision-makers (operators of highway sections) responsible for a toll collection yard operation system to be able to rationalize the activities and optimize the readiness factors, depending on the funds allocated to spare parts.

**Figure 5:**
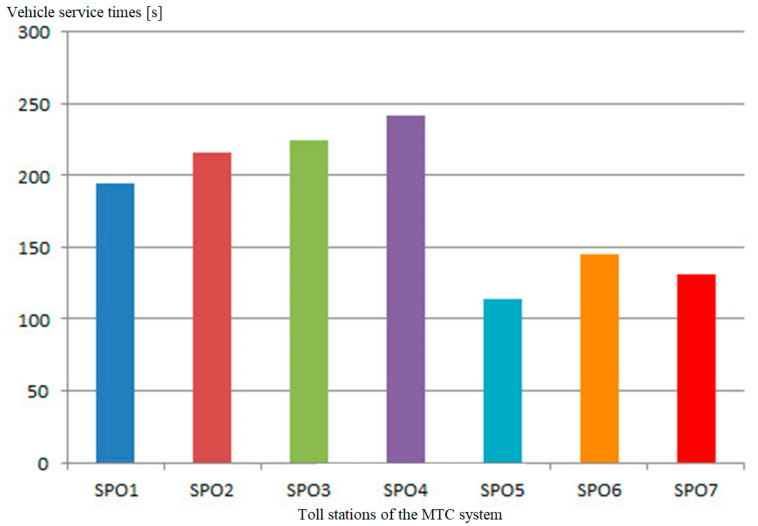
Distribution of vehicle service times at individual toll stations of the MTC system. Source: own study.

The method presented in this section of the paper was used in the application supporting the making of operational decisions of the MTC system, which is described below. The results of the application on the real MTC toll plaza are presented below. The results of parameter measurements obtained with the use of the application supporting the making of operational decisions of the MTC system in the capacity analysis and user service assessment mode are shown further in Section 3.1. The presented measurement results are presented for one exemplary measurement day. The data analysis concerned 2060 vehicles passing on a given day through a grouping of seven toll stations during the measurement hours from 16:00 to 19:00. The determined average service times at individual toll stations are close to the value of 20 s for servicing a single vehicle. For the entire group of toll collection stations, the average vehicle service time is 19.8 s. The comparison of service times in relation to the number of all vehicles shows that 72%, i.e., most vehicles, were served at the toll station with a time less than 20 s per vehicle. Vehicle service times at each of the analysed toll stations are not evenly distributed. The worst service times were recorded at the stations located in the middle of the grouping of stations (Figure 5), which results from the formation of the longest queues of vehicles for service there. The formation of long queues of vehicles causes greater fatigue of the collector operating the toll station, introducing a greater human factor in the form of an error during vehicle operation.

The longest periods of vehicle service times (Figure 5) were recorded at the fourth toll station located in the middle of the grouping of toll collection stations, which additionally may indicate the formation of the longest queues of vehicles to service there, resulting from the preferences of system users.

**Figure 6:**
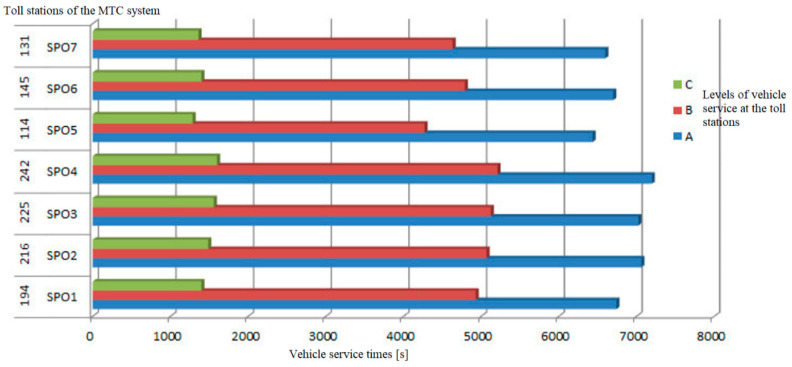
Distribution of vehicle service levels at individual toll stations of the MTC system. Source: own study.

Figure 6 shows the maximum values of service times for individual toll stations along with the periods of their service levels. For the entire cluster of seven toll stations, the occurrence of level 3 (level C in Figure 6) lasted on average for a period of 1440 s during the 3 h of measurement. This means that the waiting time of users at the grouping of seven toll collection stations of the MTC system was over 180 s for a period of 24 min within 3 h of measurement.

Taking into account the obtained measurement results, measures should be taken to shorten the waiting times at the grouping of toll collection stations. The analysed grouping of toll collection stations is a typical example of a mass service system with seven independent and parallel service stations. The process of modelling the traffic of vehicles on the motorway and their operation in the MTC system has been described using the classic problems of queuing systems. In systems of this type, in the event of system failure, there are three main methods to deal with and improve their work:

– limiting the number of vehicles,
– shorter service time,
– increasing the number of service stands in the grouping of toll collection stations.

### 3.1. Application Supporting Making Operational Decisions of the Manual Toll Collection System

**Figure 7:**
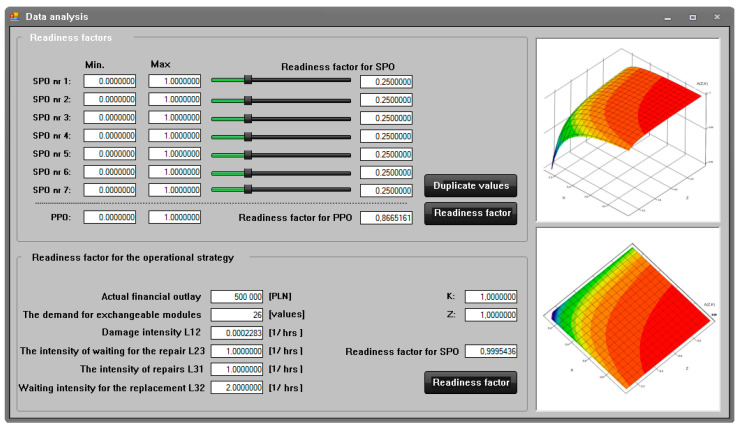
View of the system software while working in the second mode of determining the reliability indicators for the toll collection station and grouping the stations in the MTC collection. Source: own study.

The application supporting the making of operational decisions of the manual toll collection system (Figure 7) was used to determine the reliability parameters in the form of readiness indicators of the actual manual toll collection system. The reliability parameters were used to define the operational strategy of maximizing the availability ratio, taking into account the demand for replacement modules and the actual financial outlays for them in the system operation process. The following parameters are subject to registration for each toll collection station and grouping of manual toll system positions:

– the intensity of damage to the toll collection point,
– the intensity of waiting for the toll station repair,
– the intensity of repairs at the toll station,
– intensity of waiting for exchangeable modules for the toll station.

The collection of this data allows for the determination of parameters, in the form of readiness indicators of the toll collection station and grouping of positions, determining the strategy of maximizing the readiness ratio taking into account:

– demand for exchangeable modules,
– actual financial outlays allocated to them during the system operation process.

Figure 7. below presents the view of the system software working in the second mode of determining the reliability parameters of the toll collection station and grouping of stations.

The scheme of the application’s operation in the mode of determining reliability indicators for the toll collection station and grouping the stations in the manual toll collection system determines the parameters:

– availability ratio for the adopted operational strategy of the analysed toll collection station,
– readiness index for the adopted operational strategy of the analysed group of toll collection stations.

These parameters are determined based on the data recorded for each toll collection station of manual toll system positions presented above. The values indicated above by the program are used to support the management of the operation process of motorway toll collection systems, in fact, on a real toll plaza. The application of the program speeds up the process of analysing the reliability and operational data of toll stations and groups of toll stations. Thanks to this, it is possible to make rational decisions regarding the operation of these systems much faster. Additionally, the program enables the capacity analysis and evaluation of motorway user service. Along with the use of the above values of the readiness indicators for the adopted operational strategy and information on the capacity of the toll plaza and the evaluation of the service of motorway users, it is possible to quickly apply a strategy adequate to the situation for the operation of individual toll stations on the analysed plaza. This enables rational use of all stations, taking into account their operational readiness and the current process of servicing vehicles at the toll plaza. In the future, it is planned to expand the application with the functions of saving data to the database and automatically informing decision-makers responsible for the operation process about the need to purchase used exchangeable modules. Additionally, the application is planned to predict the intensity of damaging, waiting, and repairing. This will allow the system to obtain feedback about the operation and adapt itself to give the output values more accurately.

**Figure 8:**
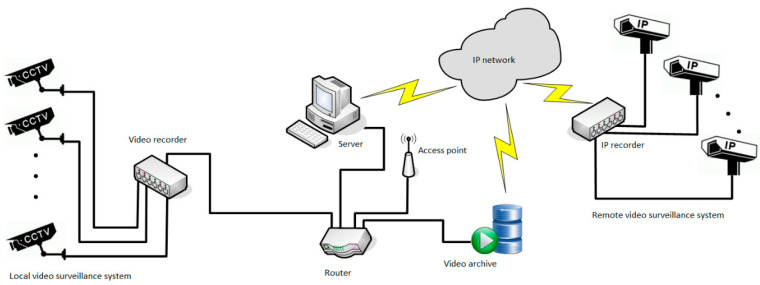
Diagram of a system supporting the process of making operational decisions of the MTC system. Source: own study.

In the second stage, the application was used to analyse the capacity and evaluate user service at a cluster of seven toll collection stations on the actual section of the motorway. The system’s capabilities allow the verification process to be carried out practically in any way and from any place, both on the basis of live (current) video material as well as previously saved and archived in the video surveillance system (Figure 8), which increases the possibilities of the control process itself and the flexibility of the entire solution, and minimizes costs—fewer operators, fewer or no business trips, etc.

In order to verify not only the toll collection process itself, but also the capabilities of the system in question, a grouping of seven toll collection stations and a time period for which the control using traditional methods has already been checked were selected for the needs of the work. In both cases, the aim is to determine the waiting time for each vehicle applying to a toll collection group. For each toll station of the MTC system, the following parameters are determined and determined:

– time of reporting (arrival) of the vehicle to the toll station,
– time of commencement of service of a given vehicle at a toll station,
– time of completion of service of a given vehicle at a toll station,
– the current length of the queue of vehicles at the toll station,
– average waiting time of the vehicle in the queue for payment,
– the average length of the queue of vehicles awaiting payment of the toll,
– average number of vehicles waiting in line for toll collection in a toll plaza.

The collection of this data allows for the determination of parameters determining the level of service in the grouping of toll collection stations:

– waiting time for service,
– length of the queue of vehicles,
– vehicle service time.

**Figure 9:**
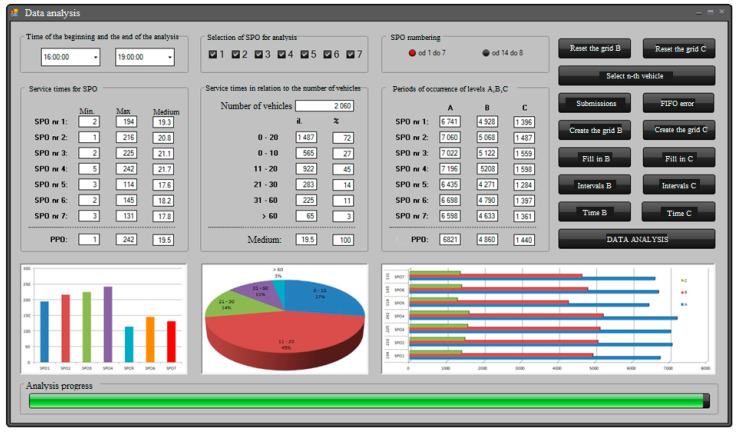
View of the system software while working in the mode of analysis related to the operation of vehicles at the toll collection station and the grouping of these tolls. Source: own study.

The above parameters made it possible to assess the levels of service of the MTC system as a grouping of seven toll collection stations on the actual section of the motorway. Figure 9 below shows the view of the system software operating in the mode of determining the above parameters related to the operation of vehicles at the toll collection station and the grouping of these tolls.

## 4. Summary and Conclusions

The effect of the application is to determine the capacity characteristics and service levels for users of the MTC system as well as the reliability parameters of the MTC system. This is to enable the decision-maker to make an adequate operational decision (in this case, the manager of a given motorway section). Limiting the number of vehicles is possible administratively or legally. However, this seems to be an unwise solution due to the universal right of drivers to access the motorway and the associated reduction in toll revenues. Reducing the volume of vehicle traffic can also be achieved by increasing the toll, however, practice shows that this is a short-term action and always poorly received by motorway users. Shortening the service time seems to be the best solution for both users and the motorway manager. In practice, however, it will be very difficult to implement, as there are limits below which this time cannot be reduced. This is due to phenomena such as vehicle delays in the queue, bad driver habits or the toll collection process itself. Some time savings can be achieved by streamlining electronic payments, better signage and directing traffic to individual toll stations, but they are unlikely to be significant. Increasing the number of service stands is certainly the most expensive solution, which results not only from the expansion of the group of toll collection stations, but also from the additional number of employees on positions.

Analyses of capacity and evaluation of vehicle servicing along with the designated operational parameters for the toll plaza make it possible to determine the optimal strategy of operation, taking into account the reliability and capacity of the system. It is extremely important, since there are often restrictions in the form of a finite resource of funding allocated to system operation. Therefore, this raises a decision-related issue: how to ensure operational continuity (system fitness) having limited funds, while simultaneously ensuring a relevant level of the readiness index and meeting the set objectives (e.g., repair cost mitigation). The developed application supporting the making of operational decisions determines the values of the readiness index for the grouping of toll collection stations, taking into account the developed operational strategy taking into account the financial outlays for inspections and the need for replacement modules. As a result, it can be used as an IT tool to support the management of the operation process of motorway toll collection systems. The application of the program speeds up the process of analysing the reliability and operation data of toll stations. Thanks to this, it is possible to make rational decisions regarding the operation of these systems much faster.

## Figures and Tables

**Table 1:** Predicting the reliability parameter values for modules of a manual toll collection system station. Source: own study based on [14].

Module Description	$\lambda \;h^{-1}$	MTBF h
Induction loop module	$10.07\times 10^{-6}$	$9.93\times 10^{4}$
Automatic passage barrier module	$4.67\times 10^{-5}$	$2.141\times 10^{4}$
Traffic signalling device module	$4.35\times 10^{-5}$	$2.299\times 10^{4}$
Driver display module	$3.56\times 10^{-6}$	$2.809\times 10^{5}$
Cash register module	$4.55\times 10^{-6}$	$2.189\times 10^{5}$
Power supply module	$7.85\times 10^{-5}$	$1.274\times 10^{4}$
Traffic lane controller module	$6.87\times 10^{-5}$	$1.456\times 10^{4}$

**Table 2:** List of the number of replaceable modules at a toll collection station for the determination of its readiness index appropriate value. Source: own study based on [14].

Number of Replaceable Modules [pcs]	$\mathrm{Readiness}\;\mathrm{Index}\;\mathrm{Value}\;A_{ST}$
1	0.9827238
2	0.9913988
13	0.9988597
26	0.9995436

**Table 3:** List of impact values of a toll collection station replaceable module demand index and the index on expenditure on replaceable modules on the value of its readiness index. Source: own study based on [14].

Φ—Actual Expenditure on Replaceable Modules	Ψ—Demand for Replaceable Modules	Readiness Index Value *A*_ST_
1	1	0.9995436
0.2	1	0.9986320
0.1	1	0.9974948

**Table 4:** List of the number of replaceable modules together with the actual expenditure on replaceable modules needed to ensure an appropriate value of a toll collection station readiness index. Source: own study based on [14].

Actual Expenditure on Replaceable Modules [PLN]	Demand for Replaceable Modules [pcs]	Readiness Index Value *A*_ST_
500,000	26	0.9995436
100,000	26	0.9986320
50,000	26	0.9974948

## Data Availability

Data sharing not applicable.
